# Application of Failure Mode and Effects Analysis to Improve the Quality of the Front Page of Electronic Medical Records in China: Cross-Sectional Data Mapping Analysis

**DOI:** 10.2196/53002

**Published:** 2024-01-19

**Authors:** Siyi Zhan, Liping Ding, Hui Li, Aonan Su

**Affiliations:** 1 Zhejiang Provincial People's Hospital Hangzhou China

**Keywords:** front page, EMR system, electronic medical record, failure mode and effects analysis, FMEA, measures

## Abstract

**Background:**

The completeness and accuracy of the front pages of electronic medical records (EMRs) are crucial for evaluating hospital performance and for health insurance payments to inpatients. However, the quality of the first page of EMRs in China's medical system is not satisfactory, which can be partly attributed to deficiencies in the EMR system. Failure mode and effects analysis (FMEA) is a proactive risk management tool that can be used to investigate the potential failure modes in an EMR system and analyze the possible consequences.

**Objective:**

The purpose of this study was to preemptively identify the potential failures of the EMR system in China and their causes and effects in order to prevent such failures from recurring. Further, we aimed to implement corresponding improvements to minimize system failure modes.

**Methods:**

From January 1, 2020, to May 31, 2022, 10 experts, including clinicians, engineers, administrators, and medical record coders, in Zhejiang People’s Hospital conducted FMEA to improve the quality of the front page of the EMR. The completeness and accuracy of the front page and the risk priority numbers were compared before and after the implementation of specific improvement measures.

**Results:**

We identified 2 main processes and 6 subprocesses for improving the EMR system. We found that there were 13 potential failure modes, including data messaging errors, data completion errors, incomplete quality control, and coding errors. A questionnaire survey administered to random physicians and coders showed 7 major causes for these failure modes. Therefore, we established quality control rules for medical records and embedded them in the system. We also integrated the medical insurance system and the front page of the EMR on the same interface and established a set of intelligent front pages in the EMR management system. Further, we revamped the quality management systems such as communicating with physicians regularly and conducting special training seminars. The overall accuracy and integrity rate of the front page (*P*<.001) of the EMR increased significantly after implementation of the improvement measures, while the risk priority number decreased.

**Conclusions:**

In this study, we were able to identify the potential failure modes in the front page of the EMR system by using the FMEA method and implement corresponding improvement measures in order to minimize recurring errors in the health care services in China.

## Introduction

The electronic medical record (EMR) system is the main carrier of medical information that has details about the whole process of a physician’s treatment for a patient [1]. The information on the front page of the EMR is condensed, which includes a patient’s basic information, disease diagnosis, information on surgical or invasive operations, and medical expenses [2]. Since January 1, 2013, almost all tertiary hospitals in China have submitted the front pages of the EMRs of inpatients to the Hospital Quality Monitoring System led by the Bureau of Medical Administration and Medical Service Supervision and National Health and Family Planning Commission of the People’s Republic of China [3]. The quality and management of the front pages of EMRs are critical for their application in medical services [4], research [2,5], education [6], and hospital management [7]. For example, some indicators for assessing the capacity of hospital medical services, such as the services for surgery and disease diagnosis, often utilize the information through the front page of the EMR for statistical purposes. However, there are many difficulties in the management of the front page of EMR. A survey conducted by the National Medical Record Management Quality Control Center of China [8] showed that more than 230 million front pages of EMRs in 2020 are established in China. Each of them contain over 100 fields. However, there are only 2.5 full-time coders on average in each hospital among 5439 medical institutions, and only 67.9% of them perform special quality control, while 24.2% of them use information technology to control the quality of the front page of the EMR system.

For reforming the medical insurance payment methods in China, the Chinese State Council’s version of health insurance issued a notice in 2019 on the issuance of technical specifications and grouping schemes for the national pilot of diagnosis-related grouping payments for diseases [9]. Therefore, the front page of an EMR needs to be uploaded on the websites of the Health and Wellness Committee and the Health Insurance Authority, which means coders need to edit a front page twice to meet the different needs of both the sectors. The former is for hospital performance evaluation and the latter is for patient health insurance payment. The introduction of this policy in 2019 increased the difficulty of medical record management.

Failure mode and effects analysis (FMEA) is a proactive risk management tool that originated in the US military in the 1940s. It is widely applicable to human, equipment, and system failure modes, as well as hardware and software programs. FMEA finds out all the potential failure modes in a system and analyzes their possible consequences by mapping the subsystems and each subprocess that makes up the process one by one in the product design stage and process design stage [10]. Thus, the advantage of FMEA is that problems can be identified and improved during the system development phase to avoid possible problems. Moreover, the costs incurred to address software defects and failures at an early stage are lower compared to those incurred to address defects at a later stage. Initially, FMEA was widely used in engineering [11], food safety management [12], financial management [13], and so on. Thereafter, with the rising demands in health care services, FMEA was used for proactive health care risk analysis. Doctors often use the EMR system to record patients’ visits. Any issue in the EMR system can affect the patient’s visit process and visit records. According to a systematic review [14], 158 studies published from 1998 to 2018 and classified under 4 categories, namely, health care process, hospital management, hospital informatization, and medical equipment and production, reported the use of FMEA in health care systems for proactive health care risk evaluation. In FMEA, the risk priority number (RPN) is calculated by giving a numerical value (scoring) for the severity, frequency, and detectability of the risks or failures, which enable risk assessment of the system [10]. An EMR system named Heren (Zhejiang Heren Technology Corporation), which is installed in many hospitals in China, is used by physicians and medical record management coders and quality controllers for filling out the front page. The purpose of this study was to identify the possible failures in the front page data of the EMR and their causes and effects and to propose specific improvement measures to minimize errors. Moreover, we aimed to compare the EMRs before and after introducing the measures to verify the efficacy of the improvement measures. For this, we reviewed previous relevant literature through PubMed, Embase, Web of Science, and Cochrane Library. During this review, we found that although FMEA has been used in some studies for improvement of some facets of EMRs, no study has used FMEA for improving the efficiency the front page of the EMR [15,16]. Thus, to the best of our knowledge, ours is the first study to apply FMEA to identify the potential failures on the front page of the EMR in China and the causes and effects of these failures and to perform a before-and-after comparison of the revised front page of the EMR.

## Methods

### Study Design

We conducted a cross-sectional study from January 1, 2020, to May 31, 2022, in Zhejiang People’s Hospital, which is one of the largest public hospitals in Zhejiang province with more than 100,000 hospital discharges per year. During the period of our research, the number of hospital discharges reached 250,774, which means the same number of front pages of EMRs needed to be filled and coded.

### Steps of FMEA

#### Assembling a Panel for FMEA

Ten experts, including clinicians, medical record coders, and hospital administrators, were invited to assess the potential risks of the EMR system in China. Since coders and quality controllers were necessary to ensure the accuracy of the front page of the EMR, only those who had been working full-time on this task for more than 5 years and who had achieved a coding accuracy rate of more than 95% and who had checked more than thousands of medical records for quality were included. Before we began our study, the organizer introduced the theme of our study to ensure that every expert knew the process of FMEA and the importance of a front page of an EMR. Then, the time and place for each discussion was planned to ensure that the process ran smoothly.

#### Mapping the Process and Subprocesses

Each expert mapped the process and subprocess of completing a front page of an EMR alone initially to avoid interference from others. For example, there are 2 data sources for the content on the front page of the EMR: information automatically imported from the hospital information system that is mainly used by physicians and information that is filled in manually by the physician. Thus, different experts could map their own process according to their work experience. Thereafter, all experts were gathered to draw the final process and subprocess to achieve the completeness of the whole system.

#### Brainstorming to Identify Potential Failure Modes in Each Subprocess and Their Causes and Effects

The implementation process of this step is consistent with the mapping process. At first, each expert could think about every potential failure mode individually. Then, all the experts summarized all the modes and discussed many more potential failure modes by brainstorming once again. In addition, the views on effects and reasons for failure modes were exchanged by experts. Since there were so many issues that could result in potential failure modes, our team summarized the main causes and created a questionnaire for randomly selected physicians and coders to answer.

#### Calculating the RPN

A scoring criterion was used to evaluate the severity, frequency, and detectability of the failures, and each dimension was divided into 10 points. Then, the RPN was calculated by using the score of the 3 dimensions (RPN = Severity × Frequency × Detectability) to evaluate the final score of each failure mode, which ranges from 0 to 1000. To improve the consistency and accuracy of scoring, the rating weight of each expert was based on their professional title grade, work experience, and familiarity with FMEA. In addition, a risk assessment criterion was established to avoid any dispute about the scores given by the experts.

#### Proposing Improvement Measures for Each Failure Mode

Since a low RPN could result from severity, frequency, or detectability and a low score for each dimension could be caused by many different reasons, it is necessary to find out the main issues. According to the Pareto principle, 80% of the consequences are due to only 20% of the potential causes [17]. Our team used the Pareto principle to identify the pressing causes that need to be addressed. Then, the experts proposed one or more corresponding improvement measures for each failure mode. Further, the feasibility and effectiveness of improvement measures were also discussed.

#### Comparing the Quality Before and After the Improvements

The experts evaluated the quality of the front page of the EMR before and after the application of the improvement measures. The RPN score was bound to improve if these improvement measures were effective.

### Ethical Considerations

This study did not involve any patient data or ethical data, and the ethics approval committee of Zhejiang Provincial People's Hospital specified that no ethics approval was required.

### Statistical Analysis

We performed statistical analyses using SPSS (version 20.0; IBM Corp). Two-sided *t* tests were performed to compare the RPNs of the front page of the EMR before and after applying the improvement measures. *P* values <.05 were considered statistically significant.

## Results

### Assembling a Panel for FMEA

Our expert panel consisted of 10 experts in 5 different fields. There were 4 physicians, 2 coders, 2 hospital administrators, 1 quality control staff, and 1 information engineer who expressed different views and opinions on the front page of the EMR.

### Mapping the Process and Subprocesses of the Front Page of EMR

**Figure 1:**
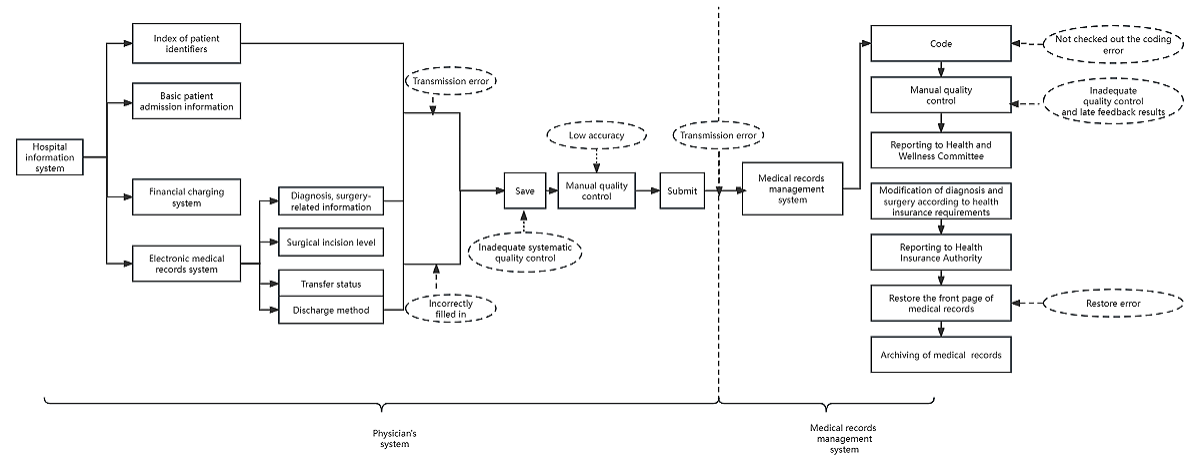
Process map of the front page of the electronic medical record system.

The expert panel identified 2 main process steps and 6 subprocesses after discussion (Figure 1). The 2 main process steps were management of the physician’s system and management of the medical record system. The 6 subprocesses were information import from physicians’ hospital information system, front page filled by physicians, front page quality control by physicians, transmission of information in the EMR system, coders’ proofreading and coding, and front page quality control by EMR management.

### Brainstorming to Identify Potential Failure Modes in Each Subprocess and Their Causes and Effects

**Figure 2:**
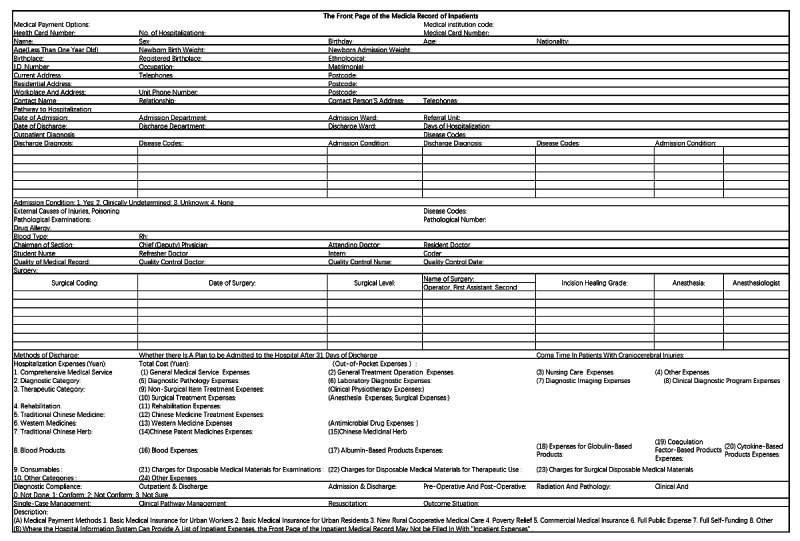
Front page of the electronic medical record.

The front page of the EMR that was evaluated in this study is shown in Figure 2. According to the process map of the front page of the EMR, the expert panel found that there were 13 potential failure modes, which can be mainly divided into 2 categories. One category is the low accuracy in a variety of information, including basic patient information, treatment information, and cost information. The other category is the low detection in a variety of information, including incorrect case header coding, incomplete quality control, and transmission errors. Regarding the causes for the failures, 115 physicians and coders filled out a questionnaire and summarized 15 main causes. The main causes were incompleteness of the front page, error in information, or incorrect diagnosis-related group, which is risky for hospital medical quality management, academic research, and medical insurance payment (Table 1).

**Table 1:** Potential failure modes with their causes and effects.

Process	Failure modes	Reasons	Effects
Transmission in the hospital information system	Basic information transmission error Inpatient information transmission error Expenses information transmission error	Data interface errors	The original data on the front page are erroneous The DRG^a^ is erroneous Affects patients’ medical reimbursement
Front page filled by physicians	Incorrectly filled-in medical information Incorrectly filled in other information	Do not understand the filling criteria Do not fill in carefully Incomplete quality control reminders	The original data on the front page are erroneous The DRG is erroneous Affects patients’ medical reimbursement
Front page quality control by physicians	Inadequate quality control Inaccurate quality control	No emphasis on quality control Unfamiliar with quality control rules Complexity of quality control rules Lack of information assistance	The original data on the front page are erroneous The DRG is wrong Affects patients’ medical reimbursement
Transmission in the physicians’ EMR^b^ system	Inconsistency between the received data in the EMR system and original data	Data interface errors Encoding conversion error	The original data on the front page are wrong The DRG is wrong
Coders’ proofreading and coding	No data errors were found Wrong code for diagnosis, surgery, or operation Restoration error Diagnostic and surgical operation codes do not meet the requirements of patients’ insurance	Formal quality control rules are too simple Lack of internal quality control reminders Insufficient professional capacity of coders Few training opportunities for coders Inadequate communication between coders and doctors The criteria are different between the requirements of patients’ insurance and front page	Erroneous data persist The DRG is wrong
Front page quality control by EMR management	Inadequate quality control Late feedback for the results of quality control	Using a sampling model to conduct quality control Insufficient professional capacity of quality control staff Complexity of quality control rules Lack of information assistance	Unable to find all errors on the front page Erroneous data persist The DRG is wrong

^a^DRG: diagnosis-related group.

^b^EMR: electronic medical record.

### Calculating the RPN

Before calculating the RPN, a risk assessment criterion was established to evaluate the quality of the front page of the EMR (Table 2).

The rating weight of each expert was calculated to reduce the influence caused by the individual subjective factors of the experts (Table 3).

**Table 2:** Risk assessment criteria for the quality management of the front page of the electronic medical record system.

Grade	Severity	Criteria for risk severity	Frequency	Criteria for risk frequency	Detectability	Criteria for risk detectability
10	Very high	Make the score of the front page of the EMR^a^ below 20	Extremely high	Every time	Very low	Cannot be detected
9	Very high	Make the score of the front page of the EMR between 20 and 30	Very high	Almost every time	Very low	Hard to detect
8	High	Make the score of the front page of the EMR between 30 and 40	Very high	One time every half day	Low	Seldom detected
7	High	Make the score of the front page of the EMR between 40 and 50	High	More than one time every day	Low	Seldom detected
6	Middle	Make the score of the front page of the EMR between 50 and 60	High	More than one time every week	Middle	Easy to be detected
5	Middle	Make the score of the front page of the EMR between 60 and 70	Middle	More than one time every month	Middle	Easy to be detected
4	Middle	Make the score of the front page of the EMR between 70 and 80	Middle	More than one time every year	High	Very easy to be detected
3	Low	Make the score of the front page of the EMR between 80 and 90	Low	One time every year	High	Very easy to be detected
2	Low	Make the score of the front page of the EMR between 90 and 100	Very low	Less than one time every year	High	Very easy to be detected
1	Very low	Does not affect the score of the front page of the EMR	Extremely low	Never	Very high	No failure modes

^a^EMR: electronic medical record.

**Table 3:** Details of the expert panel.

Position	Rating weight	Working experience (years)	Familiarity with FMEA^a^	Rating weight
Physician	High	>20	General	9/10
Physician	Middle	10-20	General	7/10
Physician	Middle	1-5	Familiar	6/10
Physician	Primary	6-10	General	5/10
Coder	High	10-20	Familiar	9/10
Coder	Middle	6-10	Familiar	7/10
Quality control staff	High	10-20	Not very familiar	7/10
Administrator	High	>20	Not very familiar	8/10
Administrator	Middle	10-20	Familiar	8/10
Information engineer	Primary	1-5	Familiar	5/10

^a^FMEA: failure mode and effects analysis.

### Proposing Improvement Measures for Each Failure Mode

**Figure 3:**
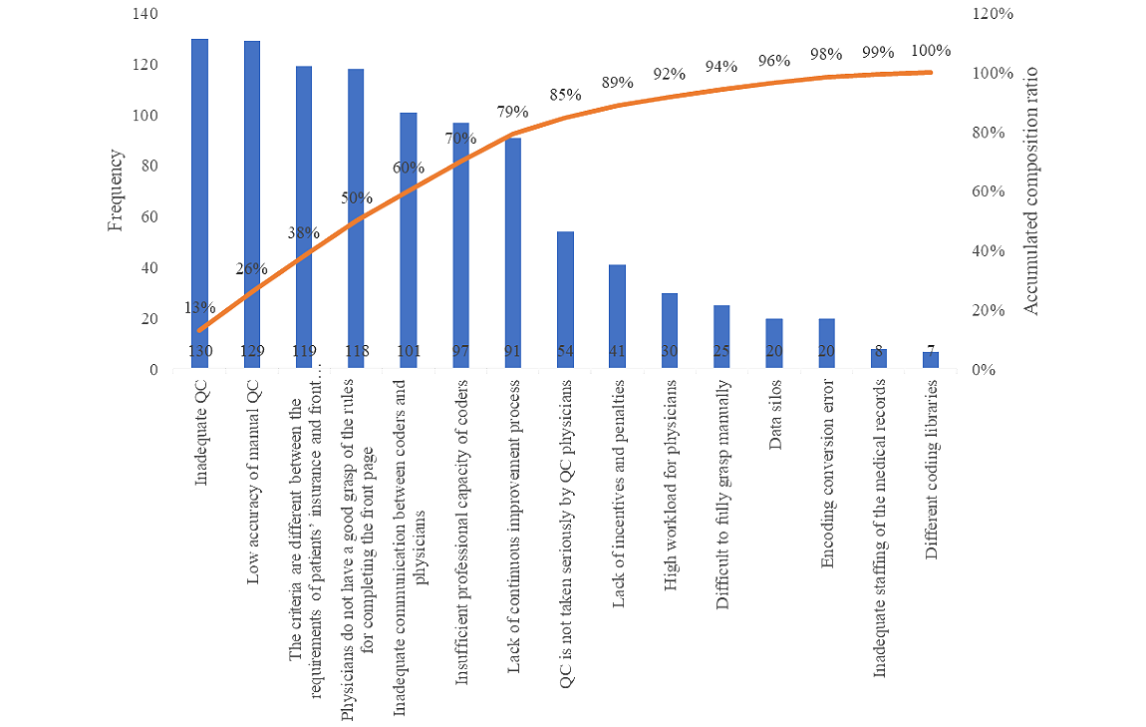
The 7 causes that contributed to 80% of the failure modes in the electronic medical record system, according to the principle of Pareto. QC: quality control.

According to the principle of Pareto, there were 7 causes in our study that contributed to 80% of the consequences (Figure 3), which can be addressed by revamping the information and quality management. For example, we integrated the medical insurance system with the front page of the EMR on the same interface and established a set of intelligent front pages for the EMR management system. In addition, we revamped the management of quality, such as communicating with physicians regularly and conducting special training seminars (Table 4).

**Table 4:** Improvement measures for the causes of potential failure modes.

	Why	What	How and When	Where	Who
Revamp the information	The rules for quality control are inadequate Low accuracy in manual quality control The criteria are different between the requirements of patients’ insurance and front page details	Establish a set of intelligent front pages in the EMR^a^ management system	Added quality control rules from May to September 2021 Embedded quality control systems into the physician’s system and front page system from July to October 2021 Integrated medical insurance system and the front page of the EMR from July to December 2021	EMR center and information center	Staffs of EMR center and information engineers
Revamp quality management	Formal quality control rules are simple Insufficient professional capacity of coders Inadequate communication between coders and physician Lack of a continuous improvement process	Improve the correctness of the front page by physician Improve the professional capacity of coders Establish effective communication channels Form a continuous improvement process	From January to May 2021 Conducted special training seminars Prepared a quality and information management manual Implemented professional training regularly Invited experts for guidance Communicated with physicians regularly Provided timely feedback to physicians on quality control results	EMR center and clinical departments	Staffs of EMR center, information engineers, and physicians

^a^EMR: electronic medical record.

### Comparing the Front Page of EMR Before and After Improvement

Before carrying out improvement measures, the highest RPN given by the experts was 296.3 for the failure mode “wrong code for diagnosis, surgery, or operation,” which was due to the quality rules being too simple, while the lowest was 50.6 for “basic information transmission error,” which was caused by wrong data interface or conversion error. On average, the final RPN was 181.2. The highest score for severity was for “wrong code for diagnosis, surgery, or operation” and the lowest was for “expense information transmission error.” The highest score for frequency was for “incorrectly filled-in medical information” and the lowest was for “expense information transmission error.” As for detectability, the highest score was for “wrong diagnosis-related group code of diagnosis, surgery, or operation,” “restoration error,” and “late feedback for quality control results.” The lowest score for detectability was for “basic information transmission error.” Our team calculated the RPN of the revised front page of the EMR after implementing the improvement measures mentioned in Table 5, and the final RPN was 95.0, which was lower than that of the original front page of the EMR (RPN=181.2).

The RPN of every failure mode decreased after implementing the improvements, and the mode for the late feedback for quality control decreased the most remarkably (Table 5). In addition, the accuracy rate of the basic information (*χ*^2^_1_=269.6; *P*<.001); inpatient information (*χ*^2^_1_=175.9; *P*<.001); diagnosis, surgery, and operation code (*χ*^2^_1_=32.9; *P*<.001); and the overall accuracy rate of the front page (*χ*^2^_1_=239.3; *P*<.001) and the integrity rate of the front page (*χ* ^2^_1_=110.4; *P*<.001) increased significantly (Table 6).

**Table 5:** Comparison of the risk analysis before and after failure mode and effects analysis model improvement.

Process, failure modes			Before FMEA^a^									After FMEA					
			Severity		Frequency		Detectability		Risk priority number		Severity			Frequency		Detectability	Risk priority number
**Information import from HIS^b^**																	
	Basic information transmission error	3.1		3.4		4.8		50.6		3.1			3.1		3.7		35.6
	Inpatient information transmission error	3.9		2.3		6.6		59.2		3.6			2.1		6.2		46.9
	Expenses information transmission error	2.4		2.1		6.6		33.3		2.2			2.0		6.0		26.4
**Front page filled by physicians**																	
	Incorrectly filled-in medical information	6.1		6.6		6.4		257.7		5.8			6.2		6.0		215.8
	Incorrectly filled-in other information	2.7		5.8		6.1		95.5		2.3			5.1		5.5		64.5
**Front page quality control by physicians**																	
	Inadequate quality control	6.5		6.4		6.4		266.2		6.0			5.1		5.2		159.1
	Inaccurate quality control	6.1		6.5		6.5		257.7		4.9			5.0		4.7		115.2
**Information import from the physician’s EMR^c^ system**																	
	Inconsistency between the received data in EMR system and the original data	3.6		3.3		5.3		63.0		3.1			3.1		4.7		45.2
**Coders’ proofreading and coding**																	
	No data errors were found	5.2		6.3		6.3		206.4		4.5			5.3		4.8		114.5
	Wrong code for diagnosis, surgery, or operation	6.7		6.6		6.7		296.3		4.6			4.5		5.0		103.5
	Restoration error	6.6		6.1		6.7		269.7		4.4			4.6		4.7		95.1
**Front page quality control by EMR management**																	
	Inadequate quality control	6.1		6.5		6.0		237.9		4.6			5.1		4.6		107.9
	Late feedback of quality control results	6.1		6.4		6.7		261.6		4.7			4.9		4.6		105.9
Average			N/A^d^		N/A		N/A		181.2		N/A			N/A		N/A	95.0

^a^FMEA: failure mode and effects analysis.

^b^HIS: hospital information system.

^c^EMR: electronic medical record.

^d^N/A: not applicable.

**Table 6:** Comparison of the accuracy and integrity of the front page of the electronic medical records before and after failure mode and effects analysis model improvement.

Items	Front pages (n)	Accuracy rate of basic information	Accuracy rate of inpatient information	Accuracy rate of diagnosis, surgery, and operation code	Overall accuracy rate of front page	Integrity rate of front page
Before	48,509	94.09	95.28	97.29	93.44	96.15
After	78,890	96.09	96.74	97.81	95.48	97.26
Chi-square *(df)*	N/A^a^	269.6 (1)	175.9 (1)	32.9 (1)	239.3 (1)	110.4 (1)
*P* value	N/A	<.001	<.001	<.001	<.001	<.001

^a^N/A: not applicable.

## Discussion

The quality of the front page of an EMR is quite important not only for hospital performance management [2] but also for insurance payments to patients [15]. Thus, it is necessary to improve the effectiveness of the front page of the EMR. There are many risk management tools for investigating the potential problems in an EMR system, such as Expert Delphi [18], scenario analysis method [19], and SWOT (strengths, weaknesses, opportunities, and threats) analysis method [20]. The advantage of Expert Delphi is that everyone's opinions are collected and that of scenario analysis is that it identifies risks by designing multiple possible future scenarios. The advantage of SWOT is that it identifies the strengths, weaknesses, opportunities, and costs of the project, thus qualitatively identifying the project risks from multiple perspectives. FMEA is a risk management tool that has most of the advantages of the above tools. FMEA can not only change the occurrence of risk from postprocessing to preemptive prevention but is also a simple and a practical risk quantification method [10]. In recent years, FMEA has been widely used in various fields, including the medical field. Studies on medical services [21], medicine distribution [22], infection control [23], and medical equipment operation and maintenance [24] have used FMEA to date.

In this study, we found potential failures existing in the EMR system of China and proposed improvement measures to solve the problems by using FMEA. Our results showed that there were 2 main processes and 6 subprocesses in the EMR system that showed 13 potential failure modes. The 2 main process steps were management of the physician’s system and management of the medical record system. The 6 subprocesses were information transmission in the hospital information system, front page filled by physicians, front page quality control by physicians, information transmission in the EMR system, coders’ proofreading and coding, and front page quality control by EMR management. This finding is similar to that reported in a study performed in Indonesia [25], wherein potential failure modes included incomplete or missing medical record files, mistakes caused by coders, and excessive code writing or upcoding [25].

According to the principle of Pareto and from questionnaire responses, we found that there were 7 causes in our study that contributed to 80% of the consequences, which can be divided into 2 aspects for the resolution of errors. One aspect was to revamp the information by establishing a set of intelligent front pages in the EMR management system to solve the problems of inadequate information and inaccurate quality control and to implement different codes of management or payment. In this study, we established quality control rules for medical records and embedded them in the system first. Accurate quality control rules are important for maintaining data quality. For example, Carlson et al [26] used quality control rules to identify common logical problems, including incomplete data, invalid values, and inconsistent data, in a clinical data set of an intensive care unit. Hart et al [27] reported >50% decrease in rejected records across patient information, service information, and financial information in 6 months by using quality rules. In addition, we integrated the medical insurance system with the front page of the EMR on the same interface. The other aspect was to revamp the management of quality by conducting multichannel trainings for doctors and coders, creating a quality and information management manual, and communicating with physicians and coders regularly. Previous studies [28-30] have shown a high rate of errors in physician coding for professional services, which can be risky in medical care services. One study [31] showed that clinicians and coders differ in their understanding of disease coding and need to communicate in a timely manner. Some of our measures are also consistent with those previously reported [25] that a hospital needs to update coding training for coders and provide guidance and validation of coding for physicians as well.

After implementing improvement measures, we found that the RPN of every failure mode decreased. The most significant decline in RPN was for the mode on the late feedback for quality control results. Many studies have proved the benefits of artificial intelligence. For example, machine learning could improve the content of medical records by identifying patients' medical information [32] or by predicting the onset of disease [33]. Therefore, we applied artificial intelligence to establish an intelligent front page for the EMR management system and then embedded it in the doctor's medical record writing interface and medical record quality control interface, which made it possible for real-time quality control of the front page. The second indicator of decline was inaccurate quality control of the front page by physicians. The original data on the front page, such as basic patient information, expenses, and surgery, are filled by physicians who decide the quality of the front page mostly [34]. After the amendments, the accuracy and integrity of the front page were both improved for those measures, which helped the diagnosis-related group to be more specific and the evaluation of the hospital performance more precise. In addition, the quality of the front page of EMR is quite important for patients. A complete front page of the medical record enables doctors to grasp important information about the patient in a short period, such as family history, allergy history, and important test results and facilitates doctors to quickly and accurately judge the patient's condition and formulate diagnosis and treatment plans, thereby reducing overmedication and even erroneous medical treatment.

Human factors engineering and user-centered designs are indispensable components of mobile health technology design and implementation [35]. Human factors emphasize human needs and capabilities as the core of the design technology system, making people the most important consideration in the design process and aiming to achieve the goal of “making machines fit people.” Regarding EMR system update, physicians, medical record coders, and quality controllers are the target users, and they will resist the technology when they believe it does not meet their expectations and needs [36]. For this reason, this study was conducted through brainstorming and questionnaires to inform the needs of physicians, coders, and others regarding the front page of the EMR system. For example, incorrectly filled-in medical information and quality control proposed by physicians, coders, and other users prompted engineers to establish a set of intelligent front pages in the EMR management system. The usability of the EMR system is evaluated by its effectiveness, efficiency, and suitability for target users. Although we did not use questionnaires to analyze the satisfaction of doctors, coders, and others with the improved EMR system, the results of FMEA showed that RPN was greatly reduced after the system was improved; thus, it can be hypothesized that the user's satisfaction with the system has been enhanced. Moreover, the overall accuracy rate of the front page (*P*<.001) and the integrity rate of the front page (*P*<.001) were significantly enhanced after implementing the improvement measures, thereby demonstrating the increase in the effectiveness of the system. The number of front pages of EMR increased from 48,509 to 78,890 with the same amount of time and labor, which proves that the efficiency of the system was also improved.

Our study has several strengths. First, medical research FMEA has mostly been performed for health care processes, hospital management, etc. For example, a study performed in Sri Lanka used FMEA to improve medication safety in the dispensing process [22], while another study aimed to increase the efficiency and success rate of patients with acute ischemic stroke [25]. No study has used FMEA for improving the front page of EMR in China before. Therefore, this is the first study performed in China, which can provide the base for future studies. Second, most studies only used FMEA to find potential failure modes and propose improvement measures, but the system was not evaluated after the application of those measures. However, our study used FMEA to compare the RPN of the front page of the EMR before and after applying the improvement measures to verify the efficiency of the system. Our study also has some limitations. The first limitation was that the method we used is not advanced since there are many better methods such as data envelopment analysis [37] and fuzzy RPN method [38]. The second limitation was that the process of scoring the system by the experts was subjective although we had set weights for the experts' scores. The third limitation was that we did not use additional methods to validate the results, which we aim to improve in the future. Lastly, although the EMR system called Heren has been used in many hospitals, different hospitals may use different types of Heren. Consequently, the generalizability of this study and the findings should be considered cautiously. In conclusion, we improved the front pages of the EMRs in China based on the potential failure modes found by the FMEA method.

## Abbreviations

EMR: electronic medical record
FMEA: failure mode and effects analysis
RPN: risk priority number
SWOT: strengths, weaknesses, opportunities, and threats

## Data Availability

Data sets are available from the corresponding author on reasonable request.
